# Editorial: The Nine Grand Challenges in Global Mental Health

**DOI:** 10.3389/fpsyt.2021.822299

**Published:** 2022-01-11

**Authors:** Malek Bajbouj, Thi Minh Tam Ta, Ghayda Hassan, Eric Hahn

**Affiliations:** ^1^Global Mental Health Section, Department of Psychiatry, Charité – Universitaetsmedizin Berlin, Berlin, Germany; ^2^Department of Psychology, Université du Québec à Montréal, Montréal, QC, Canada

**Keywords:** global health, migration, pandemic, education, health literacy, health disadvantage

Grand global challenges significantly impact mental health and well-being in vulnerable populations across the globe. Today, national health systems and infrastructures are often not sufficiently equipped to react effectively to these grand challenges' multiple and interrelated consequences. The World Health Organization has identified 13 urgent health challenges for the upcoming decade (see Table 1). From our perspective, nine of these challenges are crucial not only for global health but also for Global Mental Health.

**Table 1:** Grand challenges in global (mental) health.

	**Challenge**	**Impact for global mental health**
1	Conflict settings	Risk factors for affective disorders and posttraumatic stress disorders
2	Health disparities	General risk factor for mental disorders
3	Public trust	Strategy to increase public awareness and overcome stigma and mental health-related discrimination
4	Access to medicine	Prerequisite to provide successful diagnostic, preventive, and therapeutic interventions
5	Pandemics	Collateral damage on mental health due to measures against pandemics and potential socioeconomic burden
6	Climate crisis	Direct and indirect risk factors for mental disorders
7	New technologies	Strategy to strengthen mental health systems in LMICs
8	Education of health workers	Strategy to strengthen mental health systems in LMICs
9	Child and adolescence health	Vulnerable population
10	Dangerous products	
11	Water, sanitation, and hygiene	
12	Antimicrobial resistance	
13	Stopping infectious diseases	

## Challenge 1: Health in Conflict and Crisis Settings

Displacement due to conflict (such as in Syria, Yemen, the Central African Republic, Congo, South Sudan) or extreme violence (inflicted upon Rohingya) affected millions of people worldwide. As a direct consequence, the World Migration Report counted 272 million people as migrants (3.5% of the world's population). The global refugee population amounted to 25.9 million. The number of internally displaced people reached 41.3 million (1). These numbers are of relevance for mental health for two reasons: first, a significant proportion of refugees and migrants have been exposed to violence (2) and went through traumatic experiences [(3); Walther et al.] in their home countries or during the flight. Second, after arrival in the receiving countries, refugees, migrants, and asylum seekers experience a variety of postmigration stressors such as unclear legal status, unemployment, the absence of the core family, or the housing situation [(4); Hajak et al.]. These stressors and traumatic experiences before, during, and after flight are likely to impact incidence rates of stress-related (pooled prevalence 29.9%) and posttraumatic disorders [pooled prevalence: 39.8%; (5)].

## Challenge 2: Access to Treatment and Medical Services

Thus, it is evident that migrants, refugees, and asylum seekers are overrepresented in clinical populations with mental disorders (6). However, this phenomenon is not restricted to mental health: migrants have a higher likelihood of obesity and are often more likely to live an unhealthier lifestyle [e.g., smoking, (7)]. Against this background, it is mandatory that this vulnerable population is provided with context-sensitive and privileged access to health care facilities. However, the opposite is the case. In many regions of the world, universal health coverage (UHC) paradoxically builds multiple barriers toward and within the health care system for migrants and refugees as one of the most vulnerable groups. In addition, refugees and migrants face additional difficulties such as poor health literacy as well as language and cultural barriers. Therefore, it is surprising that in countries like Germany, migrants have a lower mortality as compared to individuals within the German non-migrant populations (8). Since this observation is likely to be due to a highly selected migrant population, it is essential to develop institutional and procedural strategies to overcome barriers. These strategies are available as illustrated by the example of a central clearing clinic (Bajbouj et al.) and resource-saving stepped and collaborative care models (9).

## Challenge 3: Public Trust and Health Literacy

The best health infrastructure and the largest mental health care resources are of no value if affected populations are stigmatized or if no knowledge exists regarding diseases or treatment options on the caretakers' side. Equally important, health literacy needs to be present on the caregivers' side with respect to cultural differences in idioms of distress or concerning culture-sensitive treatment modifications (10, 11). Two examples of interventions considering cultural aspects are in diagnostics (Lindheimer et al.) and therapy (Rayes et al.) are provided in this special issue, as well as insights into the role of stigma in the provision of mental health services in Germany (Lindheimer et al.; Bär et al.), Jordan, and the Kurdistan Region of Iraq (12, 13).

## Challenge 4: Health Disparities

Despite the higher risk of developing mental disorders, migrants and refugees seem to be at a significant disadvantage within specialized mental health care with lower rates of hospitalization, the lower likelihood for referral to mental health specialists, and higher rates of treatment discontinuation (14, 15) as well as shorter treatment duration of inpatients in psychiatric wards (Frizi et al.). The phenomenon of health disparities in health systems is universal, not specific to mental health care and general drivers of inequity such as socioeconomic status, neighborhood, and insurance status can potentially impact all medical disciplines (16).

## Challenge 5: Mental Health Care in Pandemics

The current COVID-19 pandemics have deciphered difficulties in accessing health structures, lack of public trust, and health disparities in mental health care across the globe. The pandemic served as an acute stressor for health systems and their health workers, as illustrated in quantitative and qualitative studies from Tunisia (Slama et al.), Kenya (Kwobah et al.), Pakistan (Ali et al.), and Switzerland (Weilenmann et al.). Importantly, the pandemic-related stress in healthcare workers has both short-term and considerable long-term effects on the incidence of affective and posttraumatic stress disorders (Waring and Giles). But pandemics impact mental health beyond health care workers. Beyond them, COVID-19 patients and their relatives experience high anxiety levels (Dorman-Ilan et al.). These effects seem to be pronounced in vulnerable populations such as migrants (Moran et al.) or with adverse childhood experiences (Huang et al.). For those beneficiaries, but also for health care professionals, various low-threshold interventions had been suggested to overcome such pandemic-related stress, including very basic psychosocial interventions such as exercise, mindfulness practices, religious practices, or social engagement that are preferably applied in group settings (Mashaphu et al.; Maric et al.).

## Challenge 6: Health and Climate Crisis

There is increasing evidence that climate change does have direct and indirect effects on mental health. Direct effects comprise phenomenons such as eco-anxiety or exacerbation of existing severe mental disorders by adverse environmental factors (17). Indirect effects are due to the socioeconomic impacts of climate change which in turn constitute risk factors for a variety of psychiatric syndromes and disorders such as suicidality, affective disorders, or addiction (18).

## Challenge 7: New Technologies

Across the globe, there is a need for health care strengthening and a need to address the psychological needs of patients with mental disorders (19). New technologies may provide the opportunity to overcome these by connecting beneficiaries of low- and middle-income countries to experts in better-positioned regions of the world, by broadly providing health literacy programs, low threshold interventions (20), or by educating health care workers (see challenge 8). On the other hand, new digital technologies pose a risk of substituting evidence-based mental health interventions and promoting less rigorously evaluated treatments for persons living in LMICs.

## Challenge 8: Education of Health Workers

Treatment of patients with mental disorders needs relevant, and thus context adapted human capacities in the mental health sector. To strengthen mental health systems, especially in LMICs, awareness in policymakers and planners (21) is required as well as structured training programs for mental health care providers. An evident approach to addressing this medical need is to increase the capacity to train experts of different disciplines regionally working in the field. Stigmatization of mental disorders, lack of existing training structures, and political preferences constitute significant barriers that need to be addressed to build significant capacities in this highly significant field of medicine and health care (22).

## Challenge 9: Child and Adolescence Health

29.3% of the global population is younger than 18 years. This population is especially vulnerable to external stressors resulting in immediate responses or later programming of mental disorders (23, 24). Although the need to primarily protect this subpopulation is obvious, the availability of training of respective specialists and tailored interventions is widely lacking, especially in LMICs. Thus, it constitutes a global challenge to promote population-based interventions to improve mental health in this highly vulnerable and large subpopulation. Against this background, the articles of the current Research Topic “*Global Mental Health in Times of Pandemic and Migration*” shed light on the multiple interdependencies between mediating factors of mental health, for instance, maternal health [(25), in this issue], and these global challenges and their impact on individuals, populations, and societies across the globe. The special issue brings eleven national perspectives describing specific and general problems within mental health care systems and suggests generalizable solutions addressing the nine challenges in global mental health.

## Author Contributions

MB wrote the first draft of the editorial and developed the conceptional background. TT, GH, and EH further developed the concept and extended the manuscript with relevant content. All authors contributed to the article and approved the submitted version.

## Conflict of Interest

The authors declare that the research was conducted in the absence of any commercial or financial relationships that could be construed as a potential conflict of interest. The Handling Editor WR declared a shared affiliation, though no other collaboration, with one of the authors MB, TT, and EH at the time of the review.

## Publisher's Note

All claims expressed in this article are solely those of the authors and do not necessarily represent those of their affiliated organizations, or those of the publisher, the editors and the reviewers. Any product that may be evaluated in this article, or claim that may be made by its manufacturer, is not guaranteed or endorsed by the publisher.

## References

[1] World Migration Report (2021). Available online at: https://publications.iom.int/system/files/pdf/wmr_2020.pdf

[2] Abbott A. The mental-health crisis among migrants. Nature. (2016) 538:158–160. 27734887 10.1038/538158a

[3] Arsenijević J Schillberg E Ponthieu A Malvisi L Ahmed WAE Argenziano S. A crisis of protection and safe passage: violence experienced by migrants/refugees travelling along the Western Balkan corridor to Northern Europe. Confl Health. (2017) 11:6. 28416965 10.1186/s13031-017-0107-z PMC5392386

[4] Walther L Kröger H Tibubos AN Ta TMT von Scheve C Schupp J. Psychological distress among refugees in Germany: a cross-sectional analysis of individual and contextual risk factors and potential consequences for integration using a nationally representative survey. BMJ Open. (2020) 10:e033658. 10.1136/bmjopen-2019-033658 32819926 PMC7440818

[5] Hoell A Kourmpeli E Salize HJ Heinz A Padberg F Habel U. Prevalence of depressive symptoms and symptoms of posttraumatic stress disorder among newly arrived refugees and asylum seekers in Germany: systematic review and meta-analysis. BJPsych Open. (2021) 7:e93. 10.1192/bjo.2021.54 33938425 PMC8142547

[6] Ng E Zhang H. The mental health of immigrants and refugees: Canadian evidence from a nationally linked database. Health Rep. (2020) 31:3–12. 10.25318/82-003-x202000800001-eng 32816413

[7] Statistisches Bundesamt. Bevölkerung und Erwerbstätigkeit. Bevölkerung mit Migrationshintergrund - Ergebnisse des Mikrozensus 2013. Fachserie 1, Reihe 2.2. Destatis, Wiesbaden (2014).

[8] Kohls M. Morbidity and Mortality of Migrants. Forschungsbericht/Bundesamt für Migration und Flüchtlinge (BAMF) Forschungszentrum Migration, Integration und Asyl (FZ). (2011).

[9] Böge K Karnouk C Hahn E Schneider F Habel U Banaschewski T. Mental health in refugees and asylum seekers (MEHIRA): study design and methodology of a prospective multicentre randomized controlled trail investigating the effects of a stepped and collaborative care model. Eur Arch Psychiatry Clin Neurosci. (2020) 270:95–106. 10.1007/s00406-019-00991-5 30796528

[10] Böge K Karnouk C Hahn E Demir Z Bajbouj M. On perceived stress and social support: depressive, anxiety and trauma-related symptoms in arabic-speaking refugees in Jordan and Germany. Front Public Health. (2020) 8:239. 10.3389/fpubh.2020.00239 32695739 PMC7338673

[11] Purgato M Carswell K Tedeschi F Acarturk C Anttila M Au T. Effectiveness of self-help plus in preventing mental disorders in refugees and asylum seekers in Western Europe: a multinational randomized controlled trial. Psychother Psychosom. (2021) 90:403–14. 10.1159/000517504 34350902 PMC8619740

[12] Karnouk C Böge K Hahn E Strasser J Schweininger S Bajbouj M. Psychotherapy in Jordan: an investigation of the host and syrian refugee community's perspectives. Front Psychiatry. (2019) 10:556. 10.3389/fpsyt.2019.00556 31456702 PMC6700211

[13] Kokou-Kpolou CK Moukouta CS Masson J Bernoussi A Cénat JM Bacqué MF. Correlates of grief-related disorders and mental health outcomes among adult refugees exposed to trauma and bereavement: a systematic review and future research directions. J Affect Disord. (2020) 267:171–84. 10.1016/j.jad.2020.02.026 32217217

[14] Gramaglia C Gambaro E Delicato C Di Marco S Di Tulli E Vecchi C. Pathways to and results of psychiatric consultation for patients referred from the emergency department. Are there differences between migrant and native patients? Transcult Psychiatry. (2019) 56:167–86. 10.1177/1363461518798844 30198829

[15] Durbin A Moineddin R Lin E Steele LS Glazier RH. Mental health service use by recent immigrants from different world regions and by non-immigrants in Ontario, Canada: a cross-sectional study. BMC Health Serv Res. (2015) 15:336. 10.1186/s12913-015-0995-9 26290068 PMC4546085

[16] Lavizzo-Mourey RJ Besser RE Williams DR. Understanding and mitigating health inequities - past, current, and future directions. N Engl J Med. (2021) 384:1681–4. 10.1056/NEJMp2008628 33951376

[17] Ingle HE Mikulewicz M. Mental health and climate change: tackling invisible injustice. Lancet Planet Health. (2020) 4:e128–30. 10.1016/S2542-5196(20)30081-4 32353287

[18] Cianconi P Betrò S Janiri L. The impact of climate change on mental health: a systematic descriptive review. Front Psychiatry. (2020) 11:74. 10.3389/fpsyt.2020.00074 32210846 PMC7068211

[19] Esponda GM Hartman S Qureshi O Sadler E Cohen A Kakuma R. Barriers and facilitators of mental health programmes in primary care in low-income and middle-income countries. Lancet Psychiatry. (2020) 7:78–92. 10.1016/S2215-0366(19)30125-7 31474568

[20] Kola L Kohrt BA Hanlon C Naslund JA Sikander S Balaji M. COVID-19 mental health impact and responses in low-income and middle-income countries: reimagining global mental health. Lancet Psychiatry. (2021) 8:535–50. 10.1016/S2215-0366(21)00025-0 33639109 PMC9764935

[21] Keynejad R Semrau M Toynbee M Evans-Lacko S Lund C Gureje O. Building the capacity of policymakers and planners to strengthen mental health systems in low- and middle-income countries: a systematic review. BMC Health Serv Res. (2016) 16:601. 10.1186/s12913-016-1853-0 27769270 PMC5073499

[22] Semrau M Alem A Ayuso-Mateos JL Chisholm D Gureje O Hanlon C. Strengthening mental health systems in low- and middle-income countries: recommendations from the Emerald programme. BJPsych Open. (2019) 5:e73. 10.1192/bjo.2018.90 31530325 PMC6700480

[23] Böge K Hahn E Strasser J Schweininger S Bajbouj M Karnouk C. Psychotherapy in the Kurdistan region of Iraq (KRI): preferences and expectations of the Kurdish host community, internally displaced- and Syrian refugee community. Int J Soc Psychiatry. (2021) 2021:20764021995219. 10.1177/0020764021995219 33583235 PMC8841631

[24] Sawyer SM Afifi RA Bearinger LH Blakemore SJ Dick B Ezeh AC. Adolescence: a foundation for future health. Lancet. (2012) 379:1630–40. 10.1016/S0140-6736(12)60072-5 22538178

[25] Jiang Q Gua Y Zhang E Cohen N Ohtori M Sun A. Perinatal mental health problems in rural China: the role of social factors. Front Psychiatry. (2021) 12:636875. 10.3389/fpsyt.2021.636875 34950062 PMC8688533

